# Platycodin D sensitizes *KRAS*-mutant colorectal cancer cells to cetuximab by inhibiting the PI3K/Akt signaling pathway

**DOI:** 10.3389/fonc.2022.1046143

**Published:** 2022-10-27

**Authors:** Yanfei Liu, Shifeng Tian, Ben Yi, Zhiqiang Feng, Tianhao Chu, Jun Liu, Chunze Zhang, Shiwu Zhang, Yijia Wang

**Affiliations:** ^1^ School of Integrative Medicine, Tianjin University of Traditional Chinese Medicine, Tianjin, China; ^2^ Department of Colorectal Surgery, Tianjin Union Medical Center, Tianjin, China; ^3^ Tianjin Union Medical Center, Tianjin Medical University, Tianjin, China; ^4^ Department of Radiology, The Fourth Central Hospital Affiliated to Nankai University, Tianjin, China; ^5^ Laboratory of Oncologic Molecular Medicine, Tianjin Union Medical Center, Tianjin, China

**Keywords:** Akt, cetuximab, colorectal cancer, platycodin D, resistance

## Abstract

Cetuximab is a monoclonal antibody against epidermal growth factor receptor that blocks downstream signaling pathways of receptor tyrosine kinases, including Ras/Raf/MAPK and PI3K/Akt, thereby inhibiting tumor cell proliferation and inducing cancer cell apoptosis. Owing to *KRAS* mutations, the effectiveness of cetuximab is usually limited by intrinsic drug resistance. Continuous activation of the PI3K/Akt signaling pathway is another reason for cetuximab resistance. Platycodin-D, a bioactive compound isolated from the Chinese herb *Platycodon grandiflorum*, regulates Akt in different trends based on tissue types. To investigate whether platycodin-D can sensitize *KRAS*-mutant colorectal cancer cells to cetuximab by inhibiting the PI3K/Akt signaling pathway, HCT116 and LoVo cells were treated with cetuximab and platycodin-D. LY294002 and SC79 were used to regulate Akt to further evaluate whether platycodin-D sensitizes cells to cetuximab by inhibiting Akt. Our results confirmed that platycodin-D increased the cytotoxic effects of cetuximab, including inhibition of growth, migration, and invasion, *via* downregulation of PI3K and Akt phosphorylation in HCT116 and LoVo cells both *in vitro* and *in vivo*. Given these data, platycodin-D may sensitize KRAS-mutant colorectal cancer cells to cetuximab *via* inhibition of the PI3K/Akt signaling pathway.

## Introduction

Epidermal growth factor receptor (EGFR) is a member of the human epidermal growth factor receptor (HER) family of receptor tyrosine kinases, and its abnormal expression is associated with the etiology of various cancers. When EGFR binds to its ligands, it activates the PI3K/Akt pathway, promotes the growth and proliferation of tumor cells, inhibits apoptosis, promotes invasion and metastasis, and regulates tumor angiogenesis (1). Cetuximab (CTX), a clinically recommended EGFR inhibitor, mainly binds to the extracellular ligand-binding domain of EGFR with high affinity and interferes with the binding of other endogenous ligands to EGFR, thereby inhibiting the activity of EGFR downstream signaling and tumor cell proliferation as well as inducing apoptosis in cancer cells (2). Wild-type *KRAS* is the gold standard for identifying cancer patients and is suitable for CTX therapy, which ensures that the Ras/Raf/MAPK pathway is effectively inhibited by CTX (3). Therefore, the effectiveness of CTX is limited by drug resistance due to downstream *KRAS* mutations (4). In addition, the continuous activation of Akt independent of EGFR is also an important reason for the emergence of resistance to CTX therapy (1); therefore, Akt inhibition is a strategy to sensitize cancer cells to CTX (5). As a downstream pathway of EGFR, the PI3K/Akt signaling pathway is dysregulated in many cancer cells. In addition to the direct regulation of this pathway by EGFR, various proteins affect Akt activation (1, 6). Therefore, there is an urgent need to develop new treatments targeting Akt to enhance the sensitivity of *KRAS*-mutant colorectal cancer (CRC) cells to CTX.

*Platycodon grandiflorum* saponin D (platycodin D, PD) is a triterpenoid saponin-like ingredient extracted from the Chinese herb *Platycodon grandiflorum*, with various biological effects. It exhibits immunostimulatory (7), anti-inflammatory (8), anti-obesity (9), and anti-atherosclerotic activities (10). In addition, PD exhibits anticancer effects against various cancer cell lines, mainly by inhibiting cell proliferation, inducing cell cycle arrest, and promoting apoptosis (11–15). It has been shown that PD up- or downregulates PI3K/Akt signaling in various types of cancer. For example, PD inhibits the proliferation of human glioma U251 cells by inhibiting PI3K/Akt signaling activation, reducing Akt phosphorylation, inducing apoptosis, and causing cell cycle arrest (16). In addition, PD significantly inhibits the expression of p-Akt in NCI-H460 and A549 cells in a dose- and time-dependent manner and induces autophagy in the two cell types by inhibiting the PI3K/Akt/mTOR signaling pathway (17). PD inhibits the migration, invasion, and growth of MDA-MB-231 human breast cancer cells by suppressing the EGFR-mediated Akt and MAPK pathways and by inhibiting the phosphorylation of Akt and mTOR (18). However, contradictory reports (19) have revealed that PD could promote p-Akt ubiquitination by increasing p-Akt levels. It has also been shown that PD is involved in the activation of extracellular signal-regulated kinases in hepatoma cells to trigger autophagy by upregulating the expression of p-Akt. Furthermore, PD can activate PI3K/Akt signaling in HEK-293 cells, promoting the phosphorylation of p-PI3K and p-Akt, but has no effect on the expression of PI3K and Akt (20). To the best of our knowledge, studies on cancer treatment with a combination of PD and CTX or other EGFR tyrosine kinase inhibitors (EGFR-TKIs) are still lacking.

In this study, two *KRAS*-mutant CRC cell lines, HCT116 and LoVo, were used to investigate the effect of PD treatment on the cytotoxicity of CTX *in vitro* and *in vivo*. The effect of PD on p-PI3K and p-Akt in CRC cells and whether the regulation of the PI3K/Akt signaling pathway affects CTX resistance were examined in this study. Our research provides a potentially reliable theory for the improvement of CTX chemotherapy efficacy with PD treatment.

## Materials and methods

### Reagents and antibodies

All cell culture media, trypsin, and antibiotics were purchased from Gibco (Grand Island, NY, USA), and fetal bovine serum (FBS) was purchased from Quacell (Zhongshan, China). PI3 Kinase p85 (19H8) Rabbit mAb, Phospho-PI3 Kinase p85 (Tyr458)/p55 (Tyr199) Antibody, Akt (pan) (C67E7) Rabbit mAb, Phospho-Akt (Ser473) (D9E) XP^®^ Rabbit mAb were obtained from Cell Signaling Technology (Danvers, MA, USA). Rabbit a beta-Actin (Loading Control), Goat anti-rabbit IgG/HRP, Phenylmethanesulfonyl fluoride (PMSF) and RIPA Lysis Buffer were purchased from Bioss (greater Boston, MA, USA). CTX was purchased from TargetMol (Boston, MA, USA). PD was purchased from Topscience (Shanghai, China). LY294002 and SABC-HRP Kit with Anti-Rabbit IgG (IHC&ICC) were obtained from Beyotime (Shanghai, China). Cell Counting Kit-8 (CCK-8), Rabbit anti-Ki67 Polyclonal Antibody and terminal deoxynucleotidyl transferase-mediated dUTP nick-end labeling (TUNEL) Apoptosis Detection Kit-diaminobenzidine (DAB) were purchased from absin (Shanghai, China).

### Cell lines

The *KRAS* mutant human colon cancer cell lines HCT116 and LoVo were purchased from the Shanghai Institutes for Biological Sciences, Chinese Academy of Sciences (Shanghai, China). All cells were cultured in an RPMI 1640 medium supplemented with 10% FBS, 100 μg/ml streptomycin, and 100 U/ml penicillin and incubated at 37°C in a humidified atmosphere containing 5% CO_2_.

### Measurement of cell viability

Cells were seeded in 96-well flat-bottom microtiter plates at a density of 5000 cells per well and treated with different concentrations of PD and CTX in four different groups: (1) cells without drug treatment as the control group; (2) cells treated with PD alone for 48 h; (3) cells treated with CTX alone for 48 h; and (4) cells treated with a mixture of PD and CTX for 48 h. Cell viability was measured using the Cell Counting Kit-8 (CCK-8) after treatment, according to the manufacturer’s instructions. Absorbance was measured at 450 nm using a microplate reader (Synergy HT; Bio-Tek, USA).

### Colony formation assay

For colony formation assay with monolayer cultures, cells were seeded in 6-well plates, and 300 cells/well were seeded in each experimental group for 2 weeks. After fixing in methanol for 15 min, the cells were stained with GIMSA application stain for 30 min, and the colonies were imaged and counted.

### Wound healing assay

Cells were cultured in 6-well plates to form a monolayer and serum-starved overnight. Then, the cells were scratched with a 10-μl sterile pipette tip to create an artificial scratch wound, washed three times with phosphate buffer solution (PBS), and incubated with serum-free medium for 48 h. Photographs of random fields from three replicate wells were captured using a light microscope (Nikon Corporation; Tokyo, Japan). The following equation was used to calculate percent wound closure: percent wound closure (%) = [1 − (Lt/L0)] × 100, where Lt represents the width of the scratch at time t, and L0 represents the initial scratch width.

### Cell invasion assay

Cells with a subconfluent growth density were serum-starved for 24 h. Next, the cells were trypsinized and seeded into the upper chamber of 24-well Transwell plates (8 μm pore size; Corning; NY, USA) at a density of 2×10^5^cells/ml in 200 μl of serum-free medium, and 600 μl of medium with 10% FBS was added to the lower chambers. After 24 h of incubation, the cells on the upper surface of the membrane were removed using cotton swabs, and those on the lower surface were fixed in methanol and stained with 0.1% crystal violet. The stained cells were counted under a light microscope. Images of randomly selected fields across three replicate wells were captured for analysis.

### Western blotting analysis

Cells were harvested from culture dishes and lysed in RIPA lysis buffer containing 1 mM phenylmethylsulfonyl fluoride and 1 mM protease inhibitors. The lysates were centrifuged, and the protein concentration was determined using a BCA protein assay kit (Solarbio; Beijing, China). Protein samples were suspended in the SDS loading buffer. After boiling for 10 min, cell protein samples (25μg protein/line) were separated on a 6–10% gel SDS-PAGE (Bio-Rad; CA, USA) and then transferred to Immobilon PVDF membranes(Immobilon; Darmstadt, Germany), which were then blocked with 5% skim milk for 1 h and probed with primary antibodies (1:1000 dilution in tris buffered saline with tween-20 (TBST); Akt antibody was used at 1:2000 dilution in TBST). Immunoreactive bands were visualized by enhanced chemiluminescence using horseradish peroxidase-conjugated goat anti-rabbit IgG secondary antibody (1:10000 dilution in TBST). Band density was quantified using ImageJ (Wayne Rasband, et al. Bethesda, MD, USA) and normalized to an indicated sample in an identical membrane. Each assay was performed in triplicate.

### Subcutaneous tumor model

A subcutaneous tumor model was used to evaluate the inhibitory effects of PD and CTX on the tumors *in vivo*. Briefly, 6-week-old male BALB/c nude mice were purchased from Beijing Hfkbio Co., Ltd (Beijing, China). HCT116 and LoVo cells were digested and washed twice with cold PBS to a final concentration of 2.5×10^7^ cells/ml in cold PBS. A 200-μl cell suspension was injected subcutaneously into one side of the armpit area of nude mice. When the tumor reached a certain size (4–5 mm) after 7 days, the mice were randomly grouped into four groups: control, PD, CTX, and combined groups (five mice in each group). The mean tumor volume and body weight were well balanced between the groups. The control group received an intraperitoneal injection of 200 μl normal saline every day; the CTX group was injected with 200 μl CTX (2 mg/kg); the PD group was intragastrically administered PD (20 mg/kg); and the combined group received PD plus CTX every day. The mice were sacrificed 14 days after dosing. Blood of mice in each group was collected from the retroorbital sinus for the measurement of serum biochemical indices. Tumors were removed from mice of the different treatment groups, and weight was measured using an electronic balance. All animal-related procedures were approved by the Animal Care and Use Committee of Tianjin Union Medical Center.

### Detection of serum biochemical parameters

Blood samples were centrifuged at 4500× g and 4°C for 15 min to collect the serum. Then, the serum concentrations of alanine aminotransferase (ALT) and aspartate aminotransferase (AST) were detected using respective commercial test kits (Solarbio, Beijing, China), according to the manual instructions.

### Immunohistochemical staining analysis

Tumor tissues were fixed in 10% buffered formalin. After 48 h of fixation, tissue samples were embedded in paraffin and sectioned into 4-μm-thick slices.

For immunohistochemical staining, tumor sections were dissolved in 10 mM sodium citrate solution (pH 6) after deparaffinization. Next, the samples were incubated overnight at 4°C with the primary antibody (dilution 1:200). The sections were then washed and incubated with a secondary antibody (dilution 1:100) for 2 h. The color was developed using DAB substrate kits. Images were captured using a Nikon microscope paired with the SPOT Advanced software (Diagnostic Instruments, Inc., MI, USA) (magnification, ×100). The ImageJ software was used to count the number of cells in each slice. Color deconvolution was used to determine positive/negative staining and was thresholded across all image samples.

### TUNEL staining analysis

For TUNEL staining, tumor sections were stained with terminal deoxynucleotidyl transferase-mediated dUTP nick-end labeling (TUNEL) to detect apoptotic cells using the TUNEL Apoptosis Detection Kit-DAB according to the manufacturer’s instructions. The slides were incubated with the TUNEL reaction cocktail and streptavidin-HRP reaction cocktail at 37°C separately for 2 h and 30 min in the dark and counterstained with DAB at 20°C for 30 min. Specimens were observed under a Nikon microscope (magnification, ×100). The TUNEL-positivity index was calculated by dividing the number of TUNEL-positive cells by the total number of cells.

### Hematoxylin and eosin staining

The lung, liver, and kidney tissues of sacrificed mice were fixed with 10% buffered formalin. Following 48 h of fixation, the samples were embedded in paraffin and sectioned into 4-μm-thick slices. The sections were stained with hematoxylin solution for 15 min and incubated with eosin for 3 min. The tissue structure and cell morphology were observed under a light microscope (magnification, ×100).

### Statistical analysis

All data are represented as the mean ± S.D. One-way analysis of variance was used to evaluate the significance of differences between different groups using the SPSS software (SPSS Inc., Chicago, IL, USA). *Statistical significance was set at P < 0.05*.

## Results

### Combined treatment with platycodin D increased the sensitivity of *KRAS*-mutant colorectal cancer cells to cetuximab

**Figure 1:**
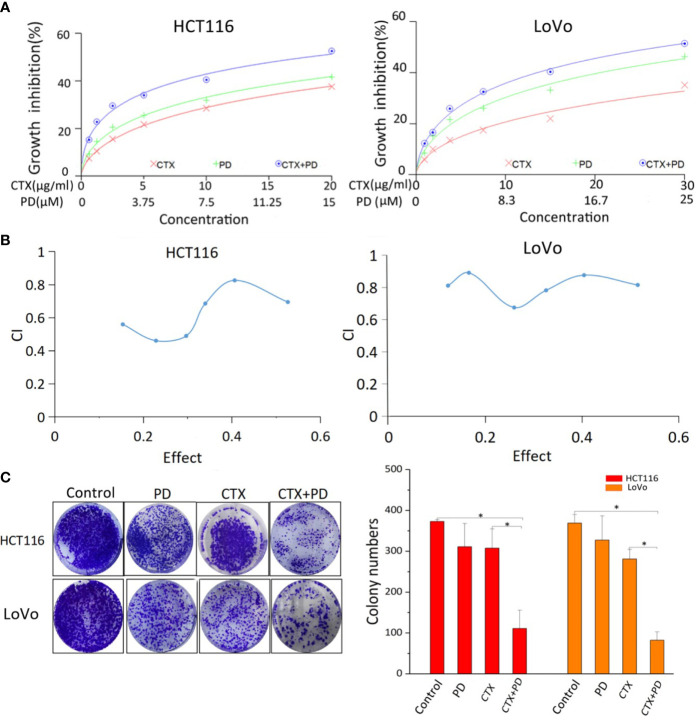
Results of CCK-8 and colony formation assays of PD- and CTX-treated cells. **(A)** The inhibitory effects of PD and/or CTX treatment in HCT116 and LoVo cells were examined by CCK-8 assay after treatment for 48 h. **(B)** The results of CCK-8 assay were analyzed with the CalcuSyn software. Combination Index (CI) was calculated by the Calcusyn software. CI < 1 was considered synergistic, CI = 1 additive, and CI > 1 antagonistic cytotoxicity. **(C)** The colony-formation assay was performed, and the results are displayed as colony numbers. Columns showed mean ± S.D. ‘Control’ was compared with the other groups, ‘CTX’ was compared with ‘CTX+PD’ using one-way ANOVA. *P < 0.05 represents a significant difference.

**Figure 1** shows the cytotoxicity of PD and/or CTX treatment on *KRAS*-mutant (HCT116 and LoVo) CRC cell lines. The cells were treated with different concentrations of PD and/or CTX for 48 h, and their viability was assessed *via* the CCK-8 assay. The results shown in **Figure 1A** indicate that both CTX and PD exert considerable cytotoxicity to the two cell lines. The IC50 of PD on HCT116 and LoVo cells was 27.62 µM and 33.78 µM, respectively, whereas the IC50 of CTX on both cell lines was 46.57 μg/ml and 105.11 μg/ml, respectively. Furthermore, combination index (CI) analysis using the CalcuSyn software showed that the combination of CTX and PD had an obvious synergistic effect on HCT116 and LoVo cells (CI<1) (**Figure 1B**).

A colony formation assay was performed to evaluate the antiproliferative effect of PD in combination with CTX. HCT116 cells were treated with 5 μM PD and 10 μg/ml CTX, and LoVo cells were treated with 6 μM PD and 20 μg/ml CTX for 48 h. The results showed that these two drugs had only a slight inhibitory effect on the cells when used alone at the indicated concentrations, but the combination treatment significantly reduced cell viability (**Figure 1C**). Taken together, the results of CCK-8 and colony formation assays both showed that PD sensitized these two cell lines to CTX. Therefore, the two drugs were used at these concentrations in all subsequent *in vitro* experiments.

### Platycodin D inhibits the PI3K/Akt signaling pathway in *KRAS*-mutant colorectal cancer cells with or without cetuximab treatment

**Figure 2:**
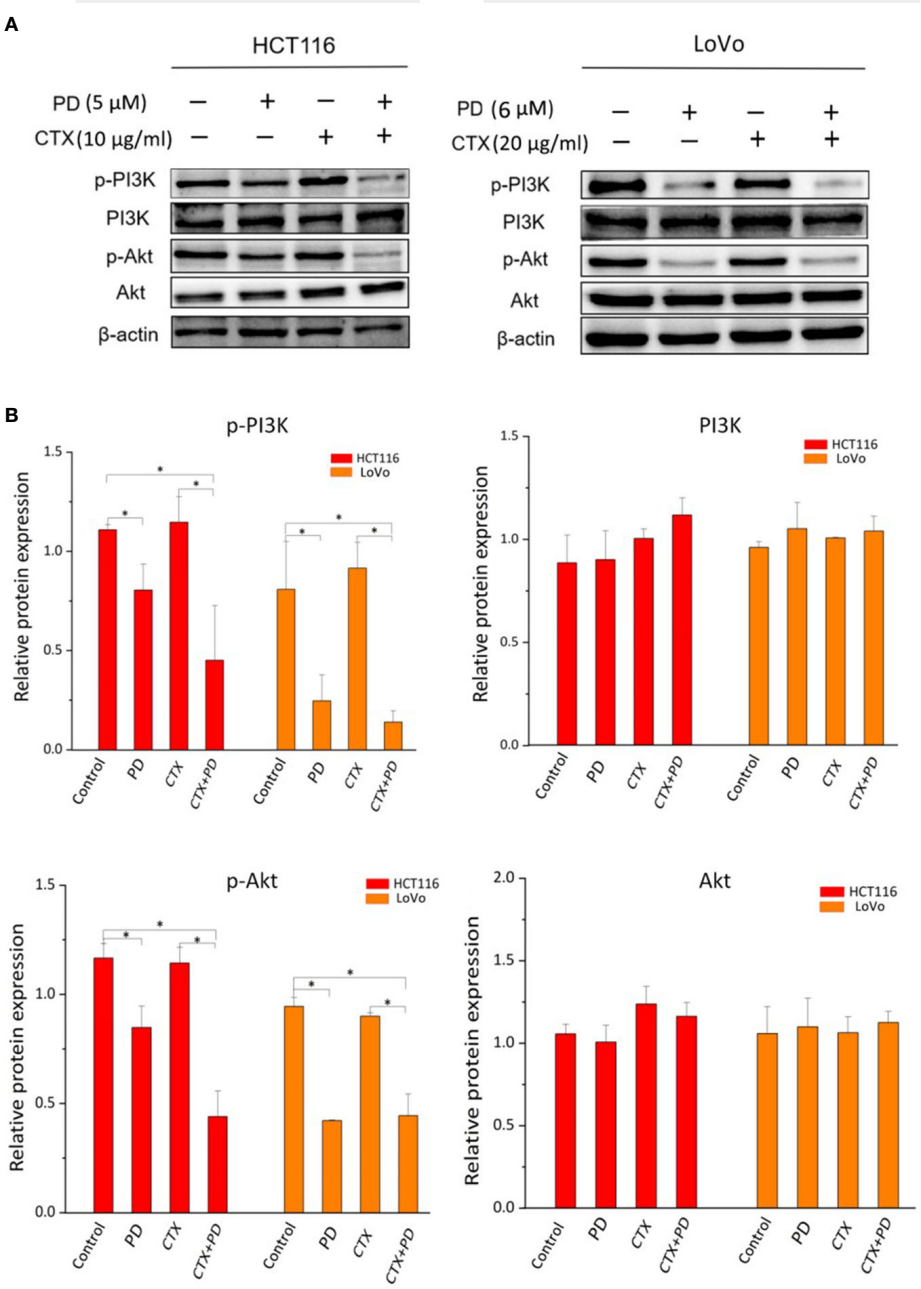
Western blotting analysis of PI3K and Akt in PD- and CTX-treated cells. **(A)** Western blot images. The left column shows protein names, including p-PI3K, PI3K, p-Akt, Akt, and β-actin, which was used as loading control. The top row shows two cell lines HCT116 and LoVo, and drug treatment with different combinations. **(B)** Bar plots of densitometric analysis. Protein expression levels are normalized to that of the loading control. Columns show the mean ± S.D. One-way ANOVA was used to analyze *P* values between groups. All groups are compared with the ‘Control’ group, the ‘CTX’ group is compared with the ‘CTX+PD’ group. *P < 0.05 represents a significant difference.

It has been reported that p-Akt is upregulated or remains unchanged after CTX treatment in some CTX-resistant cancer cells, and some drug treatments inhibit p-Akt to sensitize these cells to CTX (1, 21). PD was found to inhibit the PI3K/Akt signaling pathway and the proliferation and migration of other types of cancer cells (16). The PI3K/Akt signaling pathway is known to play an important role in the occurrence and drug resistance of cancer. Therefore, we investigated whether PD could enhance the sensitivity of *KRAS*-mutant CRC cells to CTX by acting on the PI3K/Akt signaling pathway. Western blotting analysis (**Figure 2**) indicated that CTX did not effectively decrease the phosphorylation levels of PI3K and Akt. PI3K and Akt phosphorylation in cells was significantly suppressed by a single treatment with PD or combined treatment with PD and CTX. However, there was little change in the total levels of PI3K and Akt. To further determine the role of PI3K/Akt inhibition by PD in CTX resistance, experiments were performed using LY294002 (10 µM, 48 h), a selective inhibitor specific for this signaling pathway, and SC-79 (4 µg/ml, 48 h), an Akt phosphorylation activator. These two regulators did not have detectable inhibitory effects on cells at this concentration, as confirmed by the CCK-8 assay (data not shown).

**Figure 3:**
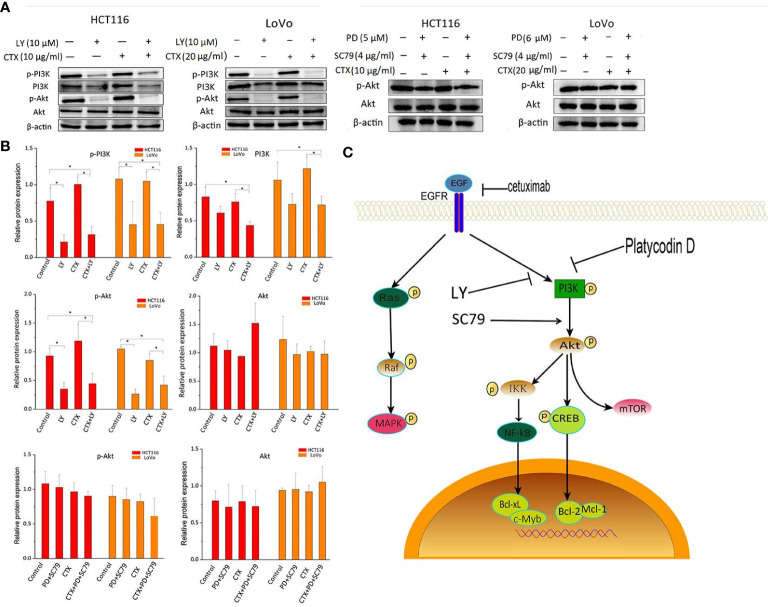
Western blot analysis of PI3K and Akt in cells treated with LY294002 and SC-79. **(A)** Western blot images. The left column shows protein names, including p-PI3K, PI3K, p-Akt, Akt, and β-actin, which was used as loading control. The top row shows two cell lines HCT116 and LoVo, and drug treatment with different combinations. ‘LY’ represents LY294002. **(B)** Bar plots of densitometric analysis. Protein expression levels are normalized to that of the loading control. Columns show the mean ± S.D. One-way ANOVA was used to analyze *P* values between groups. All groups are compared with the ‘Control’ group, the ‘CTX’ group is compared with the ‘CTX+LY’ or ‘CTX+PD+SC79’ group. *P < 0.05 represents a significant difference. **(C)** The signaling pathway by which PD enhances the cytotoxicity of CTX. A PI3K inhibitor and an Akt activator were used to confirm the inhibitory effect of PD on PI3K.

**Figure 3** shows that LY294002 decreased the phosphorylation levels of PI3K and Akt when administered alone or in combination with CTX. The combination treatment of PD and SC-79 did not decrease p-Akt effectively, suggesting that SC-79 rescued the inhibitory effect of p-Akt by PD. These results indicate that these two Akt regulators can be used to further investigate the influence of PD-induced Akt inhibition caused by PD on CTX resistance.

### Akt regulators affect cetuximab resistance

**Figure 4:**
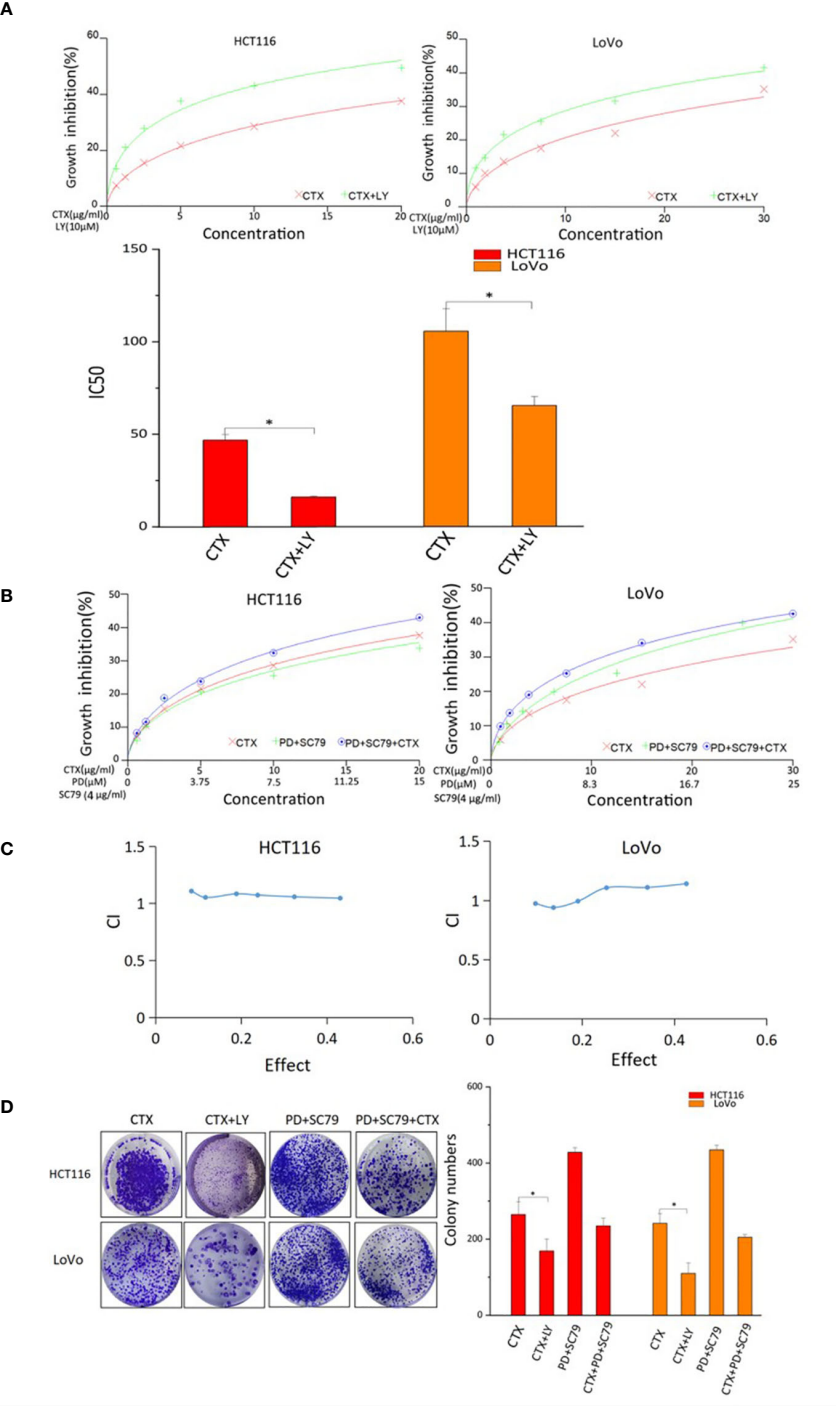
CCK-8 and colony-formation assay results of cells treated with PD, CTX, and Akt regulators. **(A)** The inhibitory effects of CTX and LY294002 treatment in HCT116 and LoVo cells were examined by CCK-8 assay after treatment for 48 h. ‘LY’ represents LY294002. The inhibitory effect of ‘CTX+LY’ is significantly higher than that of ‘CTX’ at all concentrations, as determined by one-way ANOVA (*P<*0.05). **(B)** SC79 (4 μg/ml) was administered concomitantly with PD. The inhibitory effects of PD+SC79 and/or CTX treatment in HCT116 and LoVo cells were examined by CCK-8 assay after treatment for 48 h. **(C)** The CCK-8 assay results of PD+SC79 and/or CTX treatment were analyzed with the CalcuSyn software. Combination Index (CI) was calculated by the Calcusyn software. CI < 1 was considered synergistic, CI = 1 Tadditive, and CI > 1 antagonistic cytotoxicity. **(D)** The colony-formation assay was performed, and the results are displayed as colony numbers in the column diagram. HCT116 cells were treated with 5 μM PD and 10 μg/ml CTX, whereas LoVo cells were treated with 6 μM PD and 20 μg/ml CTX. Cells were treated with 4 µg/ml SC-79 and 10 µM LY294002. ‘LY’ represents LY294002. Columns showed mean ± S.D. ‘CTX’ was compared with ‘CTX+LY’ or ‘CTX+PD+SC79’ using one-way ANOVA. *P < 0.05 represents a significant difference.

The CCK-8 assay was used to investigate the effect of PI3K/Akt signaling on CTX resistance and the synergistic effects of PD and CTX. **Figure 4A** shows that CTX-induced cell growth inhibition was enhanced by the PI3K selective inhibitor LY294002, which suggests that Akt inhibition is a critical factor in CTX sensitization. In addition, when the Akt activator SC-79 was used to offset Akt inhibition by PD, the synergistic effect between PD and CTX was lost (**Figures 4B, C**). The results of the colony formation assay (**Figure 4D**) also showed that LY294002 further inhibited the growth of cells treated with CTX, and when Akt inhibition by PD was offset by SC-79, the cell growth-inhibitory effect of PD and CTX combination decreased.

### Platycodin D and Cetuximab both inhibit the migration and invasive abilities of *KRAS*-mutant colorectal cancer cells

**Figure 5:**
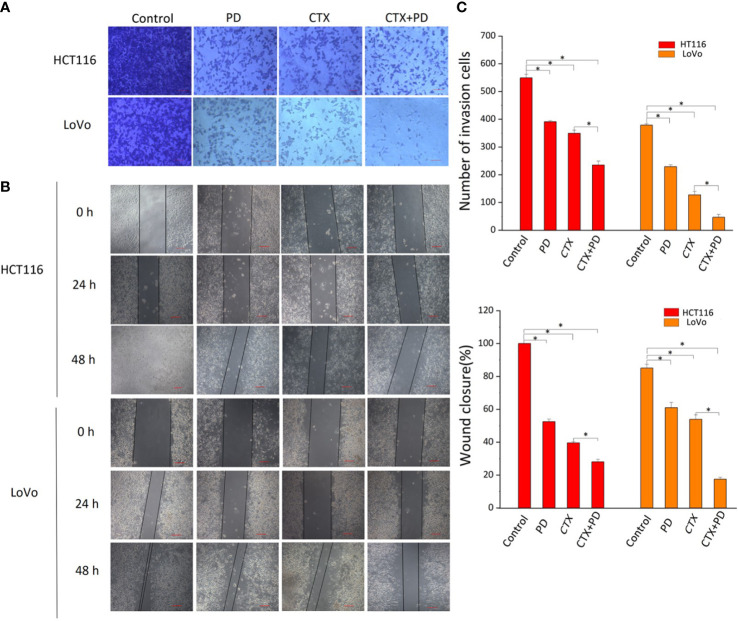
Results of Transwell and scratch wound-healing assays. **(A)** Invasion of HCT116 and LoVo cells were both measured by Transwell assay. The top row shows various drug treatments. Scale bars, 100 μm. **(B)** Migration of HCT116 and LoVo cells were both measured by scratch wound-healing assay. The top row shows various drug treatments. The left column shows the type of cells and time of measurement. **(C)** Bar diagrams of Transwell (C1) and scratch wound-healing (C2) assays. Columns show the mean ± S.D. One-way ANOVA was conducted to analyze *P* values between groups. All groups are compared with the ‘Control’ group, and the ‘CTX’ group is compared with the ‘CTX+PD’ group. *P < 0.05 represents a significant difference.

The PI3K/Akt signaling pathway contributes to the migration and invasion abilities of cancer cells during CTX treatment, and inhibition of p-Akt reduces cancer cell migration and invasion (22, 23). Considering our finding that PD inhibited p-Akt, scratch wound healing, and cell invasion assays were conducted to investigate the effects of PD and CTX treatment on the migration and invasion abilities of cancer cells, respectively. **Figure 5** shows that low concentrations of PD and CTX significantly decreased cancer cell migration and invasion, and the combination of these two drugs further decreased cancer cell migration and invasion.

### Combinative treatment with platycodin D and cetuximab exerted the strongest inhibition of tumorigenicity in *KRAS*-mutant colorectal cancer cells *in vivo*

**Figure 6:**
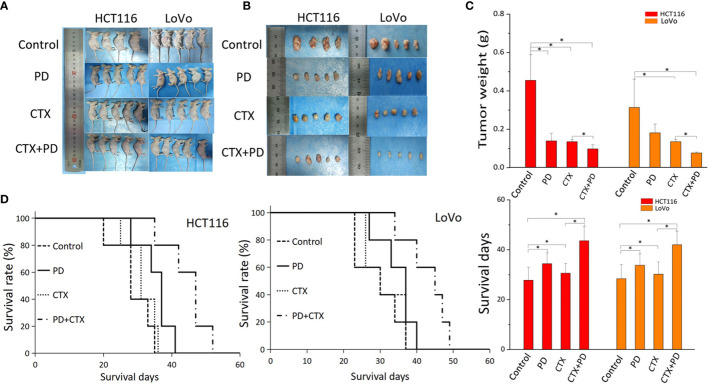
The antitumor efficacy of co-treatment with PD and CTX in subcutaneous tumor models. **(A)** Subcutaneous tumor models of HCT116 and LoVo cells were collected after the mice were sacrificed. Each group contained five mice, but one mouse of the HCT116 control group died during the experiment. **(B)** After sacrifice, subcutaneous tumors were removed and photographed. **(C)** Tumor weight was measured using an electronic balance. The mean ± S.D. is shown. One-way ANOVA was performed used to analyze P values between groups. All groups are compared with the ‘control’ group, the ‘CTX’ group is compared with the ‘CTX+PD’ group. *P < 0.05 represents a significant difference. **(D)** Survival curves of each group. Survival days of other groups are compared with those of the control group; the ‘CTX’ group is compared with the ‘CTX+PD’ group. *P<0.05 represents a significant difference.

A subcutaneous tumor model of HCT116 and LoVo cells in BALB/c nude mice was used to investigate the therapeutic efficacy of the combination treatment of PD and CTX *in vivo*. As shown in **Figure 6**, according to the comparison of the tumor weight, CTX treatment exerted considerable inhibitory effect on HCT116 and LoVo tumor growth. PD treatment only inhibited tumor growth in HCT116 cells at the statistical level. The combination of PD and CTX showed the strongest inhibitory effect among all treatments. These *in vivo* results indicated a synergistic effect of PD and CTX, similar to the *in vitro* results. Survival curves showed that all mice ultimately died owing to tumor burden. Single treatment with PD or CTX prolonged survival time compared with the control, and the combination treatment further increased survival time.

### Combination treatment of cetuximab and platycodin D decreased proliferation and enhance apoptosis *via* PI3K/Akt inhibition *in vivo*

**Figure 7:**
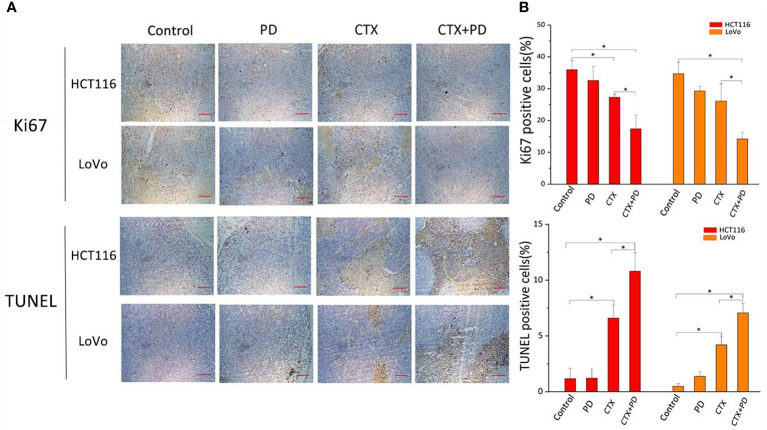
Combination treatment of CTX and PD decreases proliferation and enhance apoptosis *in vivo*. **(A)** Images of Ki67 immunohistochemical analysis and TUNEL staining assay in subcutaneous tumor models. Scale bars, 100 μm. **(B)** Quantitation of positive immunohistochemical staining for Ki67 and TUNEL staining in every group. Graph of percentage of positive cells for Ki67 and TUNEL staining (three random fields). ImageJ was used to quantify positive immunohistochemical staining results (n=3). Data are shown as the mean ± S.D. One-way ANOVA was used to analyze *P* values between groups. All groups are compared with the ‘Control’ group, and the ‘CTX’ group is compared with the ‘CTX+PD’ group. *P < 0.05 represents a significant difference.

To further investigate the mechanism by which PD sensitized CTX, we examined the expression of a cell proliferation marker (Ki-67) and the rate of apoptosis in tumor tissues from each group, as shown in **Figure 7**. Compared with that in the control groups, Ki67 expression was decreased in the CTX group, but not in the PD group, and the combination treatment further decreased the expression level of Ki67 (**Figure 7A**, Ki67 panels). To further analyze the effects of CTX and PD on tumor apoptosis, a TUNEL assay was performed on tumor samples. Histopathological staining revealed massive necrotic sheets in the combination treatment group, compared with the control group. The results of this experiment indicated a significant increase in apoptosis following the combination treatment, compared with that after single CTX treatment (**Figure 7A**, TUNEL assay panels). Furthermore, PD alone did not induce obvious tumor apoptosis. Collectively, the series of experiments revealed that the combination of CTX and PD effectively decreased tumor growth by decreasing cell proliferation (Ki67) and increasing cell death (apoptosis) in *KRAS*-mutant CRC cell lines.

**Figure 8:**
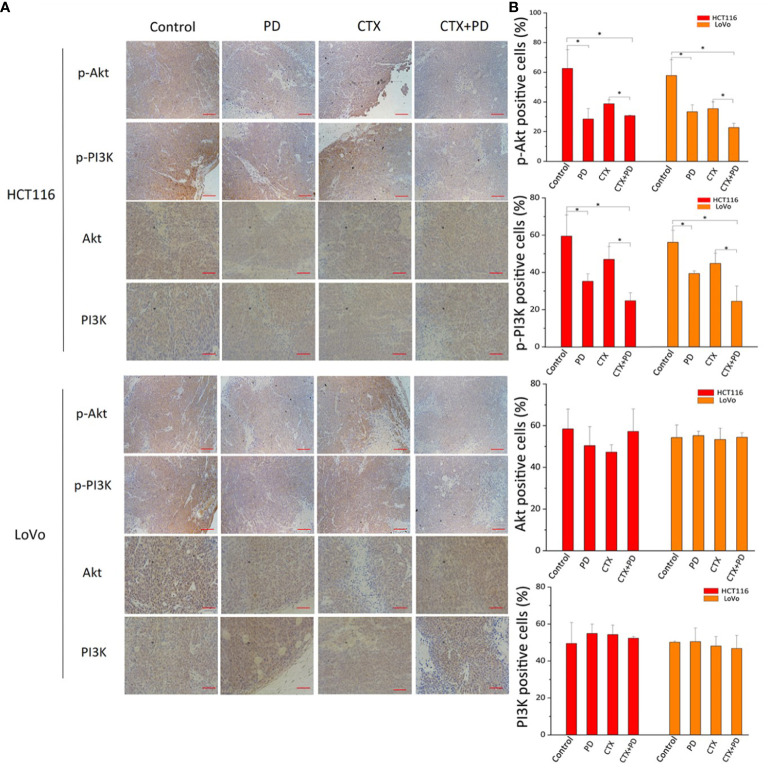
Immunohistochemical analysis of PI3K and Akt in tumor tissues treated with PD and/or CTX. **(A)** Images of immunohistochemically stained tissues. The left column shows proteins in each tumor type. The top row shows drug treatments with different combinations. Scale bars, 100 μm. **(B)** Quantitation of positive staining for p-Akt, p-PI3K, Akt, and PI3K in every group. Graph of percentage of positive cells for immunohistochemical staining (three random fields). ImageJ was used to quantify the positive immunohistochemical staining results (n=3). Data are shown as the mean ± S.D. One-way ANOVA was used to analyze *P* values between groups. All groups are compared with the ‘Control’ group, and the ‘CTX’ group is compared with the ‘CTX+PD’ group. *P < 0.05 represents a significant difference.

Next, we detected the expression of p-PI3K and p-Akt using immunohistochemical staining. As shown in **Figure 8**, the lowest expression of p-PI3K and p-Akt was found in HCT116 and LoVo subcutaneous tumors in the combination treatment group; moreover, PD treatment also significantly decreased these two phosphorylated proteins, but CTX treatment did not. These results indicated that CTX did not inhibit the PI3K/Akt signaling pathway in HCT116 and LoVo subcutaneous tumor Balb/c mouse models, and co-treatment with PD compensated for this deficiency of CTX treatment. This mechanism explains how PD sensitizes these subcutaneous tumors to CTX.

### Platycodin D decreases cetuximab-induced liver damage *in vivo*

**Figure 9:**
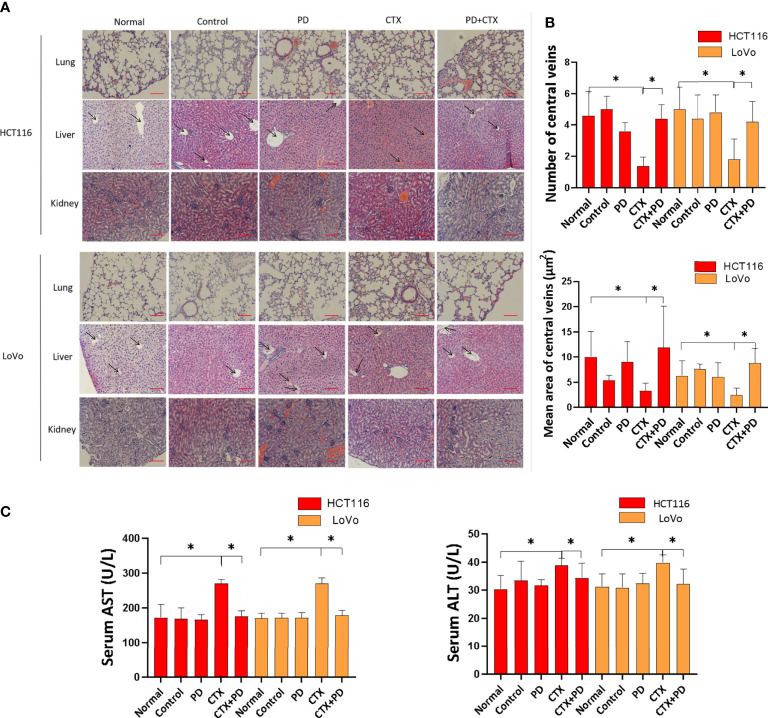
Hematoxylin and eosin staining assay of the lung, liver, and kidney. **(A)** Images of hematoxylin and eosin stained tissues. The left column shows cell types of subcutaneous tumor and types of stained tissue. The top line shows drug treatments, where ‘Normal’ represents healthy mice, and ‘Control’ represents tumor-burdened mice without drug treatment. Scale bars, 100 μm. **(B)** Quantitation of the number and vessel area of central veins in the hepatic lobules in every group. ImageJ was used to quantify the vessel diameter of central veins. Five pictures of the liver used in the column diagram were added as **Figure S3** in **Supplementary Materials**. **(C)** Concentrations of serum AST and ALT were determined in mice in different groups. Data are shown as the mean ± S.D. One-way ANOVA was used to analyze *P* values between groups. All groups are compared with the ‘Normal’ group, and the ‘CTX’ group is compared with the ‘CTX+PD’ group. *P < 0.05 represents a significant difference.

Tissue damage in the lung, liver, and kidney were analyzed by hematoxylin and eosin staining, as shown in **Figure 9**. The results indicated that PD and CTX did not exert obvious damaging effects on the lung and kidney. Furthermore, CTX decreased the number and vessel diameter of central veins in the hepatic lobules when administered alone, but these effects were reduced when CTX was administered in combination with PD (**Figure 9B**). We next investigated the serum biochemical indicators in mice. The findings revealed that the concentration of serum AST and ALT in the CTX group were increased compared with the normal group. In the combination treatment group, serum levels of AST and ALT were significantly decreased compared with the CTX group (**Figure 9C**). These data suggested that PD effectively suppressed the activities of liver functional enzymes increased by CTX in mice.

## Discussion

It is estimated that approximately 30–40% of CRC patients have *KRAS* mutation (24). Owing to *KRAS* mutations, many patients do not benefit from CTX therapy. Inhibition of Akt/PI3K, another downstream signaling pathway of EGFR, is a strategy to overcome resistance to CTX (25). Our results showed that PD reduced the CTX resistance of two *KRAS*-mutant CRC cell lines, HCT116 and LoVo. PD significantly increased the cytotoxicity of CTX when administered at a low concentrations, which only had a slight inhibitory effect on cells. Further experiments showed that Akt was not effectively inhibited by CTX in *KRAS*-mutant CRC cells, but was inhibited by PD. Therefore, two Akt regulators, LY294002 and SC79, were used to investigate whether PD could enhance CTX efficacy through Akt inhibition. As an Akt inhibitor, LY294002 was combined with CTX to determine whether Akt inhibition increased CTX efficacy. The results indicated that LY294002 enhanced CTX efficacy similar to that of PD. SC-79 is an Akt activator, and western blotting results showed that SC-79 treatment rescued Akt inhibition by PD. Therefore, the synergistic effect of SC-79 and PD combination (PD+SC-79) with CTX was evaluated by CCK-8 and colony formation assays. CI calculation indicated that although the combination treatment had the highest growth inhibition effect, PD+SC-79 had no synergistic effect with CTX. *In vitro* experiments showed that the inhibitory effect of CTX on cancer cell proliferation, migration, and invasion was negatively correlated with p-Akt levels. This implies that some downstream components of Akt that are related to migration and invasion, such as mTOR (26)or Glycogen Synthase Kinase 3β (GSK3β)/Snail (27), may be inhibited by PD. In addition, CTX itself decreases CRC cell migration and invasion to a certain degree, which may interfere with other downstream pathways of EGF/EGFR (28).

PD is a triterpenoid saponin present in the roots of *P. grandiflorum*. Increasing evidence suggests that PD has a wide spectrum of antitumor activity, exhibiting significant growth inhibitory effects and strong cytotoxicity against various cancers (29–33). Our *in vitro* and *in vivo* experiments also showed that PD exerted an antitumor effect on *KRAS*-mutant CRC cells. PD has different regulatory effects on p-Akt in various tissue types (17–20), and our *in vitro* results confirmed that low concentration of PD (about 0.2 IC50) inhibited p-Akt in *KRAS*-mutant CRC cells. This suggests that PD with low cytotoxicity can be used as an auxiliary agent to enhance CTX efficacy. *In vivo* experiments using a subcutaneous tumor model also showed that PD with a low toxic concentration had an inhibitory effect on p-Akt. The proliferation inhibition and apoptosis promotion effects were not obvious with PD treatment alone, but PD co-treatment significantly enhanced the therapeutic efficacy of CTX in subcutaneous tumors. In addition, PD had no obvious damaging effects on the lungs, liver, and kidneys of these mice, and reduced the liver damage caused by CTX treatment.

Akt inhibition contributes to antitumor effects and enhances the efficacy of antitumor drugs; therefore, many strategies involving Akt inhibition have been developed to inhibit tumor proliferation and overcome drug resistance (34, 35). In addition to acting on the Ras/Raf/MAPK pathway, CTX can exert an antitumor effect if the PI3K/Akt pathway is effectively inhibited. However, in addition to EGF/EGFR, other upstream molecules, such as integrin (36)and G protein-coupled receptor (GPCR) (37), also regulate the PI3K/Akt pathway. Therefore, the PI3K/Akt pathway may be activated by other upstream molecules owing to the feedback mechanism that occurs when EGF/EGFR is blocked by CTX. Our results showed that CTX did not effectively inhibit the PI3K/Akt pathway in *KRAS*-mutant CRC cells and that it was significantly inhibited by combination treatment with PD and CTX. This effect suggests that PD inhibited PI3K/Akt independent of EGF/EGFR to a certain degree. Therefore, the combination treatment showed tumor suppression through Akt inhibition, even in *KRAS*-mutant CRC cells.

In addition to the inhibition of downstream pathways of EGFR, CTX has other antitumor effects. For example, the interaction between CTX and the tumor microenvironment is an important factor for its therapeutic efficacy. CTX is a monoclonal antibody containing a human IgG Fc fragment. CTX triggers antibody-dependent cell-mediated cytotoxicity through Fcγ receptors in immunologic effector cells (38). Therefore, effective inhibition of Akt through combination with PD can help CTX to block the downstream pathways of EGFR, although CTX still has other antitumor effects. The combination treatment of PD and CTX may be a potential therapy for *patients with KRAS*-mutant CRC.

In this study, the potential role of PD in the sensitivity of *KRAS*-mutant CRC cells to CTX treatment was evaluated. *In vitro* and *in vivo* experimental results confirmed that PD enhanced the sensitivity of HCT116 and LoVo cells to CTX through PI3K/Akt inhibition. Our results showed that PD inhibited PI3K and Akt phosphorylation in these cells, but did not affect the expression levels of these two proteins. This conclusion will hopefully provide a prospective therapeutic strategy for patients with CRC with *KRAS* mutations.

## Data availability statement

The original contributions presented in the study are included in the article/**Supplementary Material**. Further inquiries can be directed to the corresponding authors.

## Ethics statement

The animal study was reviewed and approved by Animal Care and Use Committee of Tianjin Union Medical Center.

## Author contributions

YW, SZ, and CZ conceived and designed the experiment. YL, ST, BY, ZF, TC, and JL performed the experiments. YL and YW wrote the paper. YW, SZ, and CZ reviewed the manuscript. All authors contributed to the article and approved the submitted version.

## Funding

This work was partially supported by the Natural Science Foundation of China Grant number 81972826 and 12174203, Natural Science Foundation of Tianjin, 21JCYBJC00180, Key R&D Projects in the Tianjin Science and Technology Pillar Program, 19YFZCSY00420, and Tianjin Key Medical Discipline (Specialty) Construction Project (NO: TJYXZDXK-044A).

## Conflict of interest

The authors declare that the research was conducted in the absence of any commercial or financial relationships that could be construed as a potential conflict of interest.

## Publisher’s note

All claims expressed in this article are solely those of the authors and do not necessarily represent those of their affiliated organizations, or those of the publisher, the editors and the reviewers. Any product that may be evaluated in this article, or claim that may be made by its manufacturer, is not guaranteed or endorsed by the publisher.

## Abbreviations

EGFR, epidermal growth factor receptor; Ras, Rat sarcoma protein; Raf, Raf protein kinase; MAPK, mitogen-activated protein kinase; PI3K, phosphoinositide 3-kinase; Akt, serine/threonine kinase; PD, Platycodin-D; CTX, cetuximab; CRC, colorectal cancer; HER, human epidermal growth factor receptor; mTOR, mammalian target of rapamycin; TKIs, tyrosine kinase inhibitors; FBS, fetal bovine serum; PMSF, phenylmethanesulfonyl fluoride; CCK-8, Cell Counting Kit-8; TUNEL, terminal deoxynucleotidyl transferase-mediated dUTP nick-end labeling; DAB, diaminobenzidine; BCA, Bichinoline acid; PBS, phosphate buffer solution; TBST, Tris Buffered Saline with Tween-20; GSK3β, Glycogen Synthase Kinase 3β; EGF, Epidermal Growth Factor; GPCR, G Protein-Coupled Receptor. ALT, alanine aminotransferase; AST, aspartate aminotransferase.
