# Supplementary material for: Platycodin D sensitizes KRAS-mutant colorectal cancer cells to cetuximab by inhibiting the PI3K/Akt signaling pathway
Source: Front Oncol. 2022 Oct 27;12:1046143. doi: 10.3389/fonc.2022.1046143 (PMC9646952; doi:10.3389/fonc.2022.1046143)
Supplement: Supplementary file 1 [file DataSheet_1.docx]

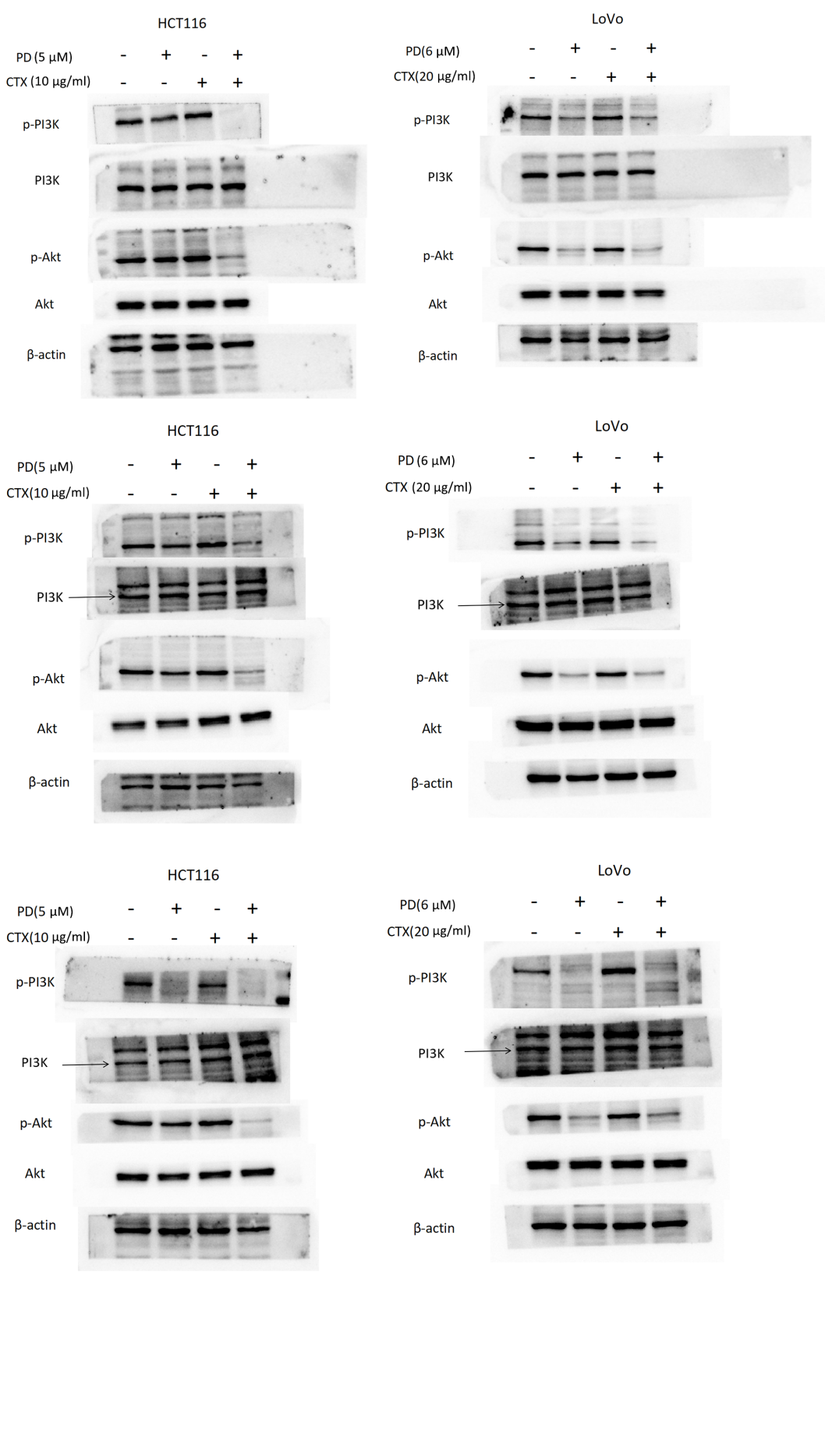


**Figure S1.** Full length blots of western blot results of Figure 2(A) .


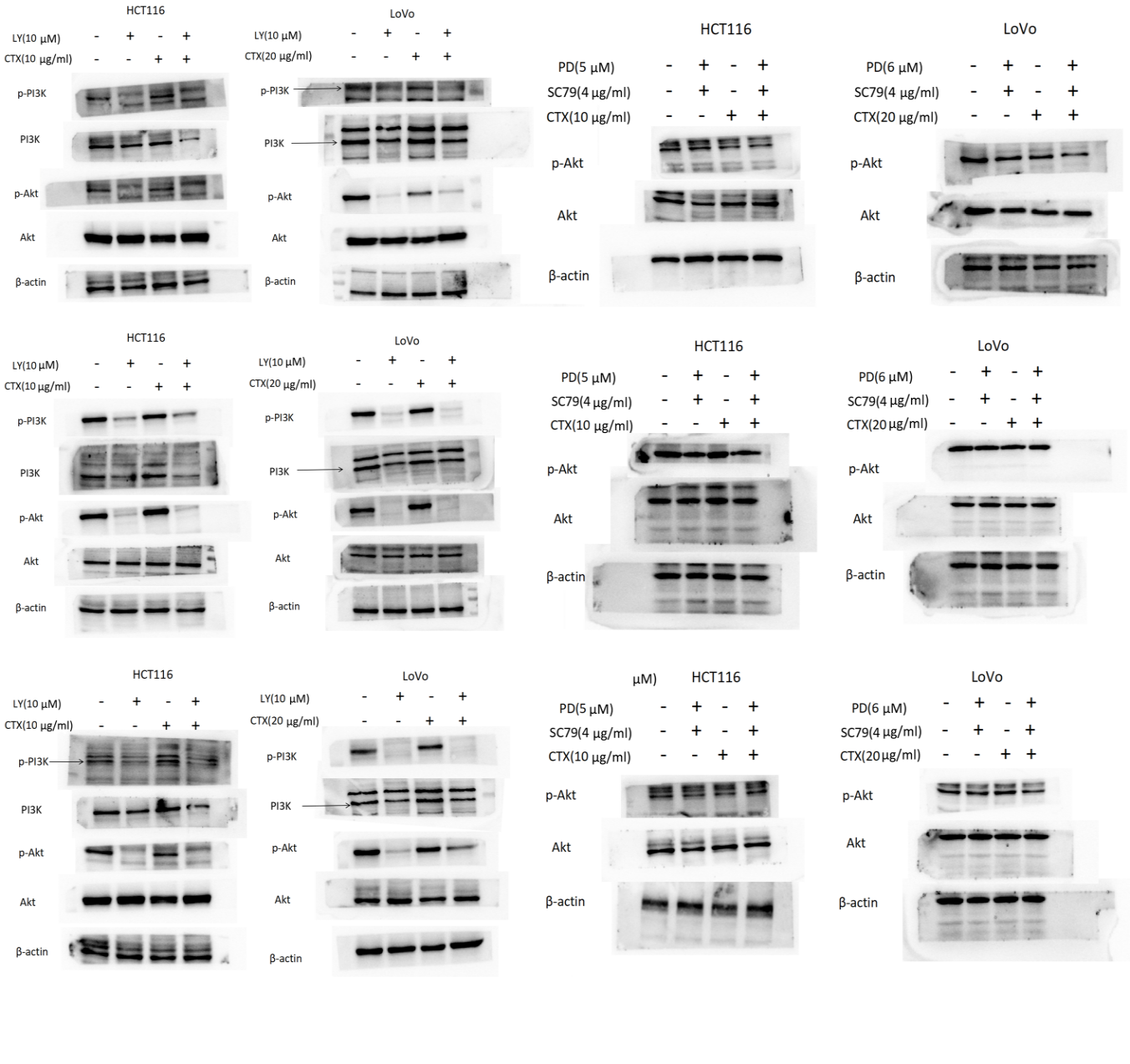


Figure S2. Full length blots of western blot results of Figure 3(A) .


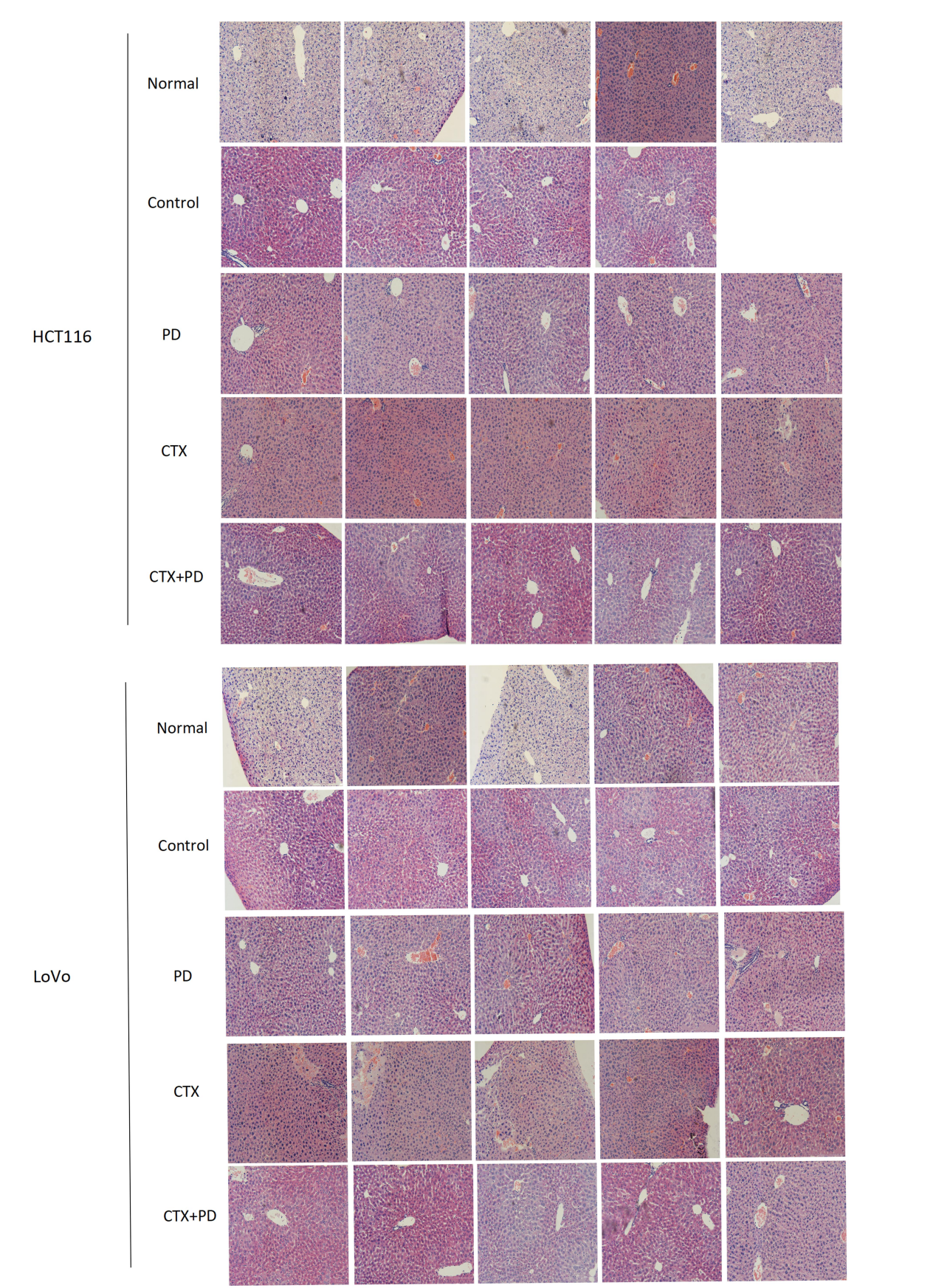


Figure S3. Five pictures of liver used in the column diagram in Figure 9.
